# Effects of Aerobic Exercise Combined with Whole-Body Electromyostimulation on Muscular, Pulmonary, and Metabolic Health in Obese Middle-Aged Women in South Korea

**DOI:** 10.3390/healthcare14070913

**Published:** 2026-04-01

**Authors:** Youn-Hwa Lee, Wi-Young So, Kyeong-Lae Kim

**Affiliations:** 1 Department of Physical Education, College of Fourth, Korea National University of Education, Cheongju 28173, Republic of Korea; lyh2100@knue.ac.kr; 2 Department of Sports Medicine, College of Humanities, Korea National University of Transportation, Chungju 27469, Republic of Korea

**Keywords:** aerobic exercise, metabolic health, metabolic syndrome, muscle function, obese middle-aged women, pulmonary function, whole-body electromyostimulation

## Abstract

**Objectives/Background**: Aerobic exercise serves as a fundamental component in improving metabolic health. However, because obese middle-aged women often find it difficult to maintain long-term exercise participation, more efficient alternative strategies, such as whole-body electromyostimulation (WB-EMS), need to be explored. This study investigated the effects of aerobic exercise combined with WB-EMS on muscle and pulmonary functioning and metabolic health indicators in obese middle-aged women in South Korea, where obesity rates have been steadily increasing. **Methods**: Women aged 40–65 years with a body mass index ≥25 kg/m^2^ who met at least three diagnostic criteria for metabolic syndrome were recruited. Of the 45 recruits, 36 completed the study. Participants were randomly assigned to a control (CON), aerobic exercise (EXG), or aerobic exercise with WB-EMS (WEG) group. The intervention consisted of a 12-week aerobic program performed three times per week for 40 min per session, with WB-EMS applied during the main exercise period. Muscle function, pulmonary function, and metabolic health indicators were assessed. Data were analyzed using two-way repeated-measures analysis of variance. Statistical significance was set at *p* < 0.05. **Results**: Significant group × time interaction effects were observed for muscle strength outcomes, with knee extensor and flexor peak torque at 60°/s (*p* < 0.001 for the right and left knee extensors; *p* = 0.001 for right knee flexors; *p* < 0.001 for left knee flexors). Post hoc comparisons indicated greater improvements in the WEG than in both the CON and EXG. Forced vital capacity (FVC) showed significant group × time interactions and time main effects (both *p* < 0.001), whereas forced expiratory volume in one second (FEV_1_) and the FEV_1_/FVC ratio showed no significant group, time, or interaction effects (all *p* > 0.05). Regarding metabolic health, BMI and waist-to-hip ratio showed significant time effects, indicating overall reductions following the intervention without significant interaction effects. Low-density lipoprotein cholesterol (*p* = 0.004) and fasting plasma glucose (*p* = 0.004) showed significant group × time interaction effects; however, post hoc analyses revealed no significant differences between the WEG and EXG. No consistent additional metabolic benefits of WB-EMS beyond aerobic exercise alone were observed. **Conclusions**: The 12-week aerobic exercise program improved muscle strength, pulmonary function, and selected metabolic indicators in obese middle-aged women. Aerobic exercise combined with WB-EMS provided additional benefits primarily for muscle strength compared with aerobic exercise alone, without conferring additional metabolic advantages.

## 1. Introduction

Advances in science and technology have increased convenience in daily life but have also reduced physical activity, thereby increasing the risk of obesity. When physical inactivity is accompanied by muscle loss and functional decline, it may lead to weight gain and a reduced basal metabolic rate [1].

Obesity is influenced by environmental, psychosocial, and genetic factors and increases the risk of various chronic diseases [2,3].

In South Korea, obesity prevalence has increased over the past decade, with women in their 50s showing the highest rates according to the Korea National Health and Nutrition Examination Survey [4]. Because obesity contributes to the national burden of chronic diseases, effective prevention strategies, including exercise interventions targeting obesity and metabolic syndrome risk, have been emphasized [5]. However, recently, obesity management has shifted toward pharmacological treatments, leading to the increased societal popularity of “diet drugs.” Glucagon-like peptide-1 receptor agonists have attracted attention based on their potent weight-loss effects, driving the rising obesity drug prescription rates in the United States since 2018 [6]. Unfortunately, these pharmacological treatments also raise concerns regarding their long-term safety and maintenance of metabolic function, as well as potential gastrointestinal side effects and muscle mass loss [7,8]. These concerns have reinforced the importance of other lifestyle-based approaches—particularly those centered on physical activity—for obesity management.

Obesity in women aged 40 years and older has been associated with functional limitations and an increased prevalence of metabolic syndrome and cardiometabolic risk factors, including elevated blood glucose, blood pressure, and body fat levels [9,10,11]. Because no single definitive treatment exists, medical and lifestyle-based interventions, including exercise, continue to be applied in parallel for obesity management [12]. Nonetheless, health management for obese middle-aged women should extend beyond simple weight reduction to address the complex health problems that accompany metabolic risk. This requires exercise programs that simultaneously aim to improve metabolic indicators—namely blood pressure, blood glucose, blood lipids, and body composition—while maintaining muscle mass and enhancing functional capacity.

Regular exercise is widely recognized as a key strategy for obesity prevention and metabolic health; however, sustained participation among obese middle-aged women remains limited because of social and physical constraints [13,14,15,16]. Several studies have demonstrated that exercise-based interventions exert beneficial effects on health outcomes in obese middle-aged women [17]. For example, a 12-week combined exercise intervention significantly increased skeletal muscle mass and reduced body fat mass, body fat percentage, and abdominal fat ratio among middle-aged women, reflecting meaningful improvements in body composition [1]. Nevertheless, such programs often require a certain level of physical fitness, time commitment, and joint loading, which may limit long-term adherence and compliance among middle-aged women with real-world restrictions.

To address these limitations, increasing attention has been directed toward technology-based exercise assistive devices. Electrical muscle stimulation (EMS) has been reported to induce exercise-like effects with relatively short application times [18]. EMS induces muscle contractions through externally applied electrical currents and has been proposed as an efficient method for enhancing muscle activation and function [15]. Electrically induced muscle contractions may promote neuromuscular adaptations, and whole-body EMS (WB-EMS) enables simultaneous activation of multiple muscle groups [19,20]. Previous studies suggest that WB-EMS may influence neuromuscular activation patterns and contribute to improvements in muscle function [21,22]. Studies in obese women have suggested that WB-EMS combined with exercise may improve body composition and cardiometabolic risk [23].

Aerobic exercise has been reported to improve blood lipid profiles in obese middle-aged women [24]. Accordingly, aerobic exercise serves as a fundamental component for improving metabolic health. Because cardiorespiratory fitness reflects integrated cardiopulmonary function, pulmonary parameters such as forced vital capacity (FVC) may provide insight into exercise-induced physiological adaptations. However, because long-term exercise adherence remains challenging for obese middle-aged women, more efficient strategies such as WB-EMS-assisted exercise may be required. Therefore, this study aimed to examine whether adding WB-EMS to aerobic exercise would produce greater improvements in muscle function and metabolic health indicators than aerobic exercise alone or a non-exercise control condition in obese middle-aged women. Pulmonary function outcomes were also assessed to explore whether exercise-induced adaptations might extend to cardiopulmonary function. Based on previous findings, it was hypothesized that aerobic exercise combined with WB-EMS would lead to greater improvements in muscle function, pulmonary function, and metabolic health indicators compared with aerobic exercise alone and the control condition.

## 2. Materials and Methods

### 2.1. Study Design

This study employed a three-group pre–post experimental design to examine the effects of aerobic exercise with and without whole-body electromyostimulation (WB-EMS) on muscle function, pulmonary function, and metabolic health-related parameters in obese middle-aged women. The overall study procedure consisted of pre-intervention education, baseline assessments, a 12-week exercise intervention, post-intervention assessments, and data analysis. During the pre-intervention education session, participants were informed in detail about the study objectives, intervention protocols, outcome measurements, and safety considerations to ensure adequate understanding. Baseline assessments were conducted to evaluate muscle function, pulmonary function, and metabolic health-related parameters and to confirm baseline homogeneity among the groups.

### 2.2. Participants

Before conducting the study, ethical approval was obtained from the Institutional Review Board of Korea National University of Education, Cheongju, Republic of Korea (approval number: KNUE-202507-SB-0513-01; approval date: 11 August 2025). Participants were recruited from urban areas in Chungcheongnam-do, Republic of Korea, using offline recruitment notices posted at local community centers and fitness facilities, as well as online announcements distributed through social networking services. Recruitment followed a convenience sampling approach. Eligible participants were Korean middle-aged women aged 40–65 years with a BMI ≥ 25 kg/m^2^ who voluntarily agreed to participate after receiving a detailed explanation of the study purpose, procedures, and potential risks. Written informed consent was obtained from all participants prior to enrollment. No financial incentives were provided for participation; however, participants received free health and fitness assessments as part of the study. A priori sample size estimation was conducted using G*Power software (version 3.1.9.4) based on the three-group repeated-measures design of the study (non-exercise control, aerobic exercise, and aerobic exercise combined with WB-EMS). Assuming a medium effect size (f = 0.25), an alpha level of 0.05, and a statistical power of 0.80 for a two-way repeated-measures ANOVA (three groups × two time points), the analysis indicated that a minimum sample size of 42 participants was required. The three groups were as follows: (1) a non-exercise control group (CON), consisting of obese middle-aged women who did not participate in any exercise intervention; (2) an aerobic exercise group (EXG), who performed the aerobic exercise program without wearing the WB-EMS device; and (3) an aerobic exercise group (WEG), who performed the identical aerobic exercise program while wearing the WB-EMS device. A total of 45 participants were initially recruited, and 36 participants were included in the final analysis after nine withdrew during the intervention period.

### 2.3. Outcome Measurements

#### 2.3.1. Muscle Function

Lower-limb isokinetic muscle function was assessed using an isokinetic dynamometer (Humac Norm, CSMi, Stoughton, MA, USA). Knee extensor and flexor muscles were evaluated to determine strength, power, and muscular endurance. The dynamometer was adjusted according to the manufacturer’s standardized protocol to accommodate each participant’s anthropometric characteristics. Participants sat with their thighs stabilized using straps, and their lateral femoral epicondyle aligned with the axis of rotation of the dynamometer. The leg being tested was secured to the knee/hip pad with the ankle maintained in a dorsiflexed position. The range of motion of the knee joint was set from full extension (0°) to 90° of flexion. A correction for gravity was applied to all measurements. Prior to testing, participants performed two to five submaximal practice trials to familiarize themselves with the testing procedure. Angular velocity conditions were selected according to the purpose of each measurement. Maximal muscle strength was assessed at 60°/s with five consecutive repetitions of the knee extension and flexion. Muscle power and muscular endurance were evaluated at 180°/s with 15 consecutive repetitions for each movement. A 1 min rest period was provided between velocity conditions to minimize fatigue. Peak torque (Nm) was used as an indicator of maximal muscle strength, average power (W) was used to assess muscle power, and total work (J) was used as an indicator of muscular endurance. The representative values (peak or mean values) automatically calculated by the dynamometer were used for statistical analysis. Peak torque and average power were normalized to body weight for analysis, while absolute values are presented.

#### 2.3.2. Pulmonary Function

Pulmonary function was assessed using a portable spirometer (Pony FX, COSMED, Rome, Italy) to comprehensively evaluate ventilatory capacity and respiratory muscle function. The primary pulmonary parameters included forced vital capacity (FVC), forced expiratory volume in one second (FEV_1_), and the FEV_1_/FVC ratio. These indices are widely used as reliable indicators of lung volume, airway resistance, and respiratory muscle performance. The measurement protocol followed standardized spirometry guidelines published by the American Thoracic Society and the European Respiratory Society [25]. Participants sat upright while wearing a nose clip and were instructed to inhale maximally and then exhale forcefully in a continuous effort. Each parameter was measured at least three times, and the highest value was used for analysis.

#### 2.3.3. Metabolic Health–Related Parameters

Metabolic health-related parameters included body composition, anthropometric measurements, blood pressure, and blood metabolic markers. Body composition variables—body weight, skeletal muscle mass, fat mass, BMI, body fat percentage, and basal metabolic rate—were assessed using bioelectrical impedance analysis (InBody 720, Biospace, Seoul, Republic of Korea). Waist and hip circumferences were measured using a non-elastic measuring tape following World Health Organization guidelines. Waist circumference was measured at the midpoint between the lowest rib and the iliac crest, and hip circumference was measured at the widest portion of the buttocks. The waist-to-hip ratio (WHR) was calculated as an index of abdominal obesity. Resting blood pressure was measured using an automated sphygmomanometer (BPBIO320, InBody, Seoul, Republic of Korea). Venous blood samples were collected following an overnight fast of at least 12 h. Participants were instructed to avoid vigorous physical activity for 24 h prior to testing and to rest for at least 30 min before blood collection. Blood samples were obtained from the antecubital vein, and all sampling and analyses were conducted by a certified clinical laboratory (S Medical Foundation). Fasting plasma glucose (FPG) was analyzed using the hexokinase method, triglycerides (TGs) using the glycerol kinase–glycerol phosphate oxidase method, and high-density lipoprotein cholesterol (HDL-C) using the GHOP/PAP method. Low-density lipoprotein cholesterol (LDL-C) was calculated using the Friedewald formula [26]. Fasting plasma glucose (FPG), triglycerides (TGs), HDL-C, and LDL-C were analyzed using an automated biochemical analyzer (Cobas 8000 e602, Roche Diagnostics GmbH, Mannheim, Germany).

### 2.4. Exercise Intervention and WB-EMS Protocol

The exercise intervention was conducted three times per week for 40 min per session over a 12-week period and consisted of warm-up, main exercise, and cool-down phases. The program was designed based on an aerobics routine for middle-aged women and included repetitive whole-body rhythmic movements performed to music, primarily involving lower-limb and hip-dominant movements. Exercise intensity was individually prescribed using the Karvonen formula based on heart rate reserve (HRR), with a target intensity ranging from 35% to 70% HRR [27]. Participants wore wrist-based heart rate monitors (Polar RS400sd, Polar Electro, Kempele, Finland) during the exercise sessions to ensure adherence to the prescribed intensity range. All exercise sessions were conducted under the supervision of a certified exercise professional to ensure proper technique, adherence to the prescribed intensity, and participant safety. The WB-EMS device was worn by participants in the WEG during the main exercise session. Stimulation frequency was progressively increased to minimize early fatigue and facilitate neuromuscular adaptation. Stimulation frequency was set at 30–40 Hz during weeks 1–4, increased to 50–60 Hz during weeks 5–8, and further increased to 65–80 Hz during weeks 9–12. In the WEG, WB-EMS parameters were applied and monitored by trained personnel throughout each session to maintain standardized delivery and safety. The detailed components and structure of the 12-week aerobic exercise program with and without WB-EMS are summarized in Table 1. Exercise session attendance was recorded throughout the intervention period to monitor participant adherence. In addition, participants were continuously monitored for any adverse events or discomfort during the exercise sessions.

### 2.5. Statistical Analysis

All statistical analyses were performed using SPSS version 26.0 (IBM Corp., Armonk, NY, USA). Descriptive statistics were calculated as means and standard deviations. The normality of data distribution was assessed using the Shapiro–Wilk test, and homogeneity of variances was evaluated using Levene’s test. While most variables satisfied the assumptions of normality and homogeneity of variances, violations of homogeneity were observed in percent body fat and systolic blood pressure; therefore, Welch’s analysis of variance was applied for these variables. To examine changes in muscle function, pulmonary function, and metabolic health-related parameters, a two-way repeated-measures analysis of variance was conducted with time (pre- and post-intervention) as the within-subject factor and group (CON, EXG, and WEG) as the between-subject factor. When significant interaction effects were observed, simple main effect analyses and Bonferroni post hoc comparisons were performed. The level of statistical significance was set at *p* < 0.05.

## 3. Results

### 3.1. Participant Characteristics and Study Flow

A total of 45 participants were initially recruited. During the intervention period, nine participants withdrew due to personal reasons unrelated to the intervention, resulting in 36 participants included in the final analysis. Baseline characteristics of the participants are presented in Table 2. No significant differences were observed among the groups at baseline. The mean attendance rate during the 12-week intervention period was 92%, and no adverse events related to the intervention were reported.

### 3.2. Muscle Function

Comparisons of lower-limb isokinetic muscle function before and after the 12-week intervention are presented in Table 3. At an angular velocity of 60°/s, significant group × time interaction effects were observed for peak torque of both knee extensors and flexors in the right and left limbs (all *p* < 0.01). Post hoc analyses indicated that the WEG demonstrated greater improvements compared with the EXG and CON, while the EXG also showed greater improvements than the CON. At an angular velocity of 180°/s, knee extensor average power showed significant group × time interaction effects in both limbs (*p* < 0.05), with post hoc comparisons indicating greater improvements in the WEG compared with the EXG and CON. In contrast, knee flexor average power did not show significant interaction or main effects. For muscular endurance assessed by total work at 180°/s, no significant interaction effects were observed for knee extensors. However, a significant group × time interaction effect was detected for left knee flexor endurance (*p* < 0.05), with post hoc analysis indicating greater increases in the EXG and WEG compared with the CON. Overall, among the muscle function variables showing significant group × time interactions, post hoc comparisons indicated that greater improvements in the WEG compared with the EXG were observed only in specific outcomes, whereas several other variables showed comparable improvements between the two exercise groups.

### 3.3. Pulmonary Function

Comparisons of pulmonary function parameters before and after the 12-week intervention are presented in Table 4. For FVC, a significant group × time interaction effect was observed (*p* < 0.001). However, Bonferroni post hoc comparisons did not reveal significant pairwise differences between groups. This suggests that the observed improvement in FVC occurred across groups rather than being attributable to a specific intervention. A significant main effect of time was also identified for FVC, indicating an overall increase following the intervention. In contrast, FEV_1_ and the FEV_1_/FVC ratio did not show significant interaction, time, or group effects. Although FVC decreased in the control group and increased in the exercise groups, these directional changes did not reach statistical significance in the post hoc analysis (*p* < 0.05).

### 3.4. Metabolic Health Indicators

Comparisons of body composition, lipid profiles, glycemic control, and blood pressure before and after the intervention are presented in Table 5. BMI and WHR showed significant main effects of time (*p* < 0.05), indicating overall improvements across the intervention period, whereas no significant group × time interaction effects were observed. Regarding lipid profiles, TG and HDL-C did not demonstrate significant interactions or main effects. In contrast, LDL-C showed a significant group × time interaction effect (*p* < 0.01), suggesting differential responses among groups over time. For glucose metabolism, FPG also demonstrated a significant group × time interaction effect (*p* < 0.01), although no significant main effects of time or group were observed. For blood pressure variables, SBP showed significant main effects of group and time, whereas the interaction effect was not significant. Diastolic blood pressure did not show significant interaction or main effects.

## 4. Discussion

### 4.1. Effects on Muscle Function

The study results showed that both aerobic exercise alone (EXG) and aerobic exercise combined with WB-EMS (WEG) improved lower-limb muscle function; however, a significantly greater improvement in the WEG compared with the EXG was observed only for knee extensor peak torque at 60°/s. Specifically, isokinetic peak torque increased significantly in both knee extensors and flexors, whereas muscle power and muscular endurance showed significant improvements only in the extensors. Although changes in the flexors did not reach statistical significance, there was a modest increasing trend, suggesting that different components of muscle function may respond differently depending on muscle use patterns and stimulus characteristics. While post hoc analyses did not always clearly differentiate between the groups (b = c) for some variables, most muscle function outcomes showed no significant differences between the EXG and WEG.

As both groups demonstrated improvements, these changes are likely attributable primarily to the effects of the exercise intervention itself; however, the additional influence of WB-EMS may have contributed selectively under certain conditions. However, since the study tests did not assess neuromuscular mechanisms directly, this interpretation is based on possibilities suggested by previous research. For example, prior studies have reported that WB-EMS can induce increases in muscle strength and lean body mass even under low mechanical loading conditions, as well as increases in cross-sectional muscle areas [28]. WB-EMS combined with functional exercise has also been shown to improve not only strength but also performance-related outcomes, such as running and jumping ability [29]. Taken together, these findings suggest that the extensor-dominant improvements observed in this study may be partially associated with the additional neuromuscular stimulus provided by WB-EMS, although such effects were not consistently observed across all variables.

WB-EMS may contribute to increases in maximal lower-limb power, potentially through the simultaneous activation of multiple motor units and the recruitment of fast-twitch muscle fibers [30]. However, as neuromuscular activation patterns were not directly assessed in this study, these mechanisms should be interpreted cautiously and are inferred from previous research. WB-EMS has also been characterized as repeatedly activating the same muscle fibers during exercise, leading to transient fatigue accumulation that may, over time, promote the body’s adaptation, improving fatigue resistance and work capacity. The significant increases in extensor muscle power and endurance observed are consistent with this type of adaptation. Conversely, the relatively limited changes in the flexors may be attributable to the exercise structure, in which extensor-dominant movements were the primary motor tasks.

Although the intervention was categorized as aerobic exercise, it consisted largely of lower-limb–dominant movements such as lunges, step exercises, and knee lifts. Repeated execution of these movements may have provided sufficient cumulative contractile stimuli to contribute to basic strength and endurance adaptation. When electrical stimulation is applied to movements with a high extensor demand, there may be greater increases in extensor activation, which aligns with the larger improvements in extensor power and endurance observed among those in the WEG. Consistent with this, previous studies have reported that lower-limb-focused combined exercise elicits greater improvements in leg strength than aerobic exercise alone does [31,32], supporting the notion that the present intervention, although aerobic in classification, incorporated meaningful repetitive muscular loading.

Overall, the improvements in muscle function observed in both groups indicate that aerobic exercise played a major role, while the addition of WB-EMS may have provided a selective benefit for specific outcomes such as knee extensor strength at 60°/s. Nevertheless, because electromyography, muscle oxygenation, blood flow, and muscle metabolic variables were not directly measured, only limited mechanistic conclusions are possible. Future studies should aim to quantify neuromuscular activation patterns and the persistence of body adaptation. Despite these limitations, the findings suggest that aerobic exercise combined with WB-EMS may serve as a low-load, high-efficiency intervention capable of improving muscle function in middle-aged women without substantially increasing joint stress. Importantly, such improvements in muscle function, achieved with minimal mechanical burden, may facilitate sustained exercise participation and thereby support long-term obesity management in populations for whom conventional high-load exercise is difficult to maintain.

### 4.2. Partial Adaptation in Pulmonary Function

The study results show that FVC increased significantly, although the FEV_1_ and the FEV_1_/FVC ratio did not exhibit significant changes. This pattern suggests that pulmonary volume and airflow-related indices may adapt asynchronously during short-term exercise programs, rather than indicating their limited efficacy. Specifically, aerobic exercise, with or without WB-EMS, may contribute to auxiliary respiratory muscle activation and improvements in thoracic mobility, leading to increases in lung volume.

The FEV_1_/FVC ratio is strongly influenced by factors such as age, sex, and body size, and minimal changes have been reported even in healthy adults following short-term interventions [33,34]. Moreover, this study’s exercise intervention did not include direct respiratory muscle training, but relied on indirect stimulation through lower-limb–dominant aerobic exercise combined with WB-EMS. In contrast, interventions that directly target respiratory muscles have demonstrated short-term improvements in FEV_1_/FVC or forced expiratory flow parameters [35], suggesting that the body’s adaptation differs according to the stimulus used.

Pulmonary function was assessed to determine whether exercise-induced improvements in body composition could translate into functional respiratory adaptations in obese individuals, who may present mechanical ventilatory constraints. Although pulmonary function is not typically altered by exercise in the general healthy population, aerobic training has been shown to improve lung volumes and ventilatory parameters in sedentary adults [36] and to exert beneficial effects on pulmonary function in clinical populations with respiratory limitations [37]. Changes in body composition may also indirectly influence pulmonary function, particularly in populations with obesity-related mechanical restrictions. Obesity has been associated with reduced diaphragmatic mobility and decreased chest wall compliance [36], and the reductions in body weight and body fat observed in the study may have contributed to improved thoracic expansion and increased FVC. Accordingly, the observed improvement in FVC may reflect secondary adaptations related to reduced mechanical load and enhanced ventilatory efficiency rather than a direct structural modification of pulmonary tissue.

Previous reports also indicate that combined interventions incorporating respiratory muscle activation and thoracic mobility stimuli are more effective for improving pulmonary function than aerobic exercise alone is [38]. Given that the present intervention involved aerobic exercise, such effects may explain the improvement in FVC, regardless of the use of WB-EMS. As large cohort studies have emphasized that gradual declines in pulmonary function are associated with increased cardiovascular risk [39], this underscores the importance of preserving pulmonary capacity rather than focusing solely on short-term absolute changes.

As such, the increase in FVC in this study may reflect an early adaptive response contributing to the maintenance of pulmonary function, rather than a comprehensive improvement in expiratory airflow. Future studies incorporating indices such as FEF_25–75_, maximal voluntary ventilation, and maximal inspiratory and expiratory pressures are warranted to more precisely characterize subtle respiratory adaptations.

### 4.3. Improvements in Metabolic Health Indicators

The study results showed that both exercise groups demonstrated significant improvements in BMI, WHR, LDL-C, FPG, and SBP. Because no significant between-group differences were observed, these changes are more likely attributable to the overall effects of the exercise intervention rather than to the additional effect of WB-EMS. Reductions in BMI and WHR suggest increased energy expenditure associated with repeated aerobic movements involving large muscle groups, which may have contributed to favorable changes in abdominal fat distribution. Consistent with this, previous studies have reported reductions in body weight, body fat percentage, waist circumference, and increases in trunk muscle activation following WB-EMS or neuromuscular electrical stimulation interventions [40].

The significant reduction in FPG may be related to enhanced glucose utilization within the skeletal muscle. Exercise has been suggested to activate pathways related to glucose uptake, such as the AMPK–AS160–GLUT4 signaling cascade, which may enhance glucose transport in skeletal muscle [41]. However, because these molecular pathways were not directly measured in the present study, the interpretation of these mechanisms should be considered hypothetical and based on evidence from previous studies. This may partially compensate for impaired metabolic regulation associated with estrogen decline in middle-aged women [42], and aligns with the observed improvements in glycemic control.

Reductions in LDL-C may also be associated with changes in fatty acid oxidation and metabolic efficiency induced by regular aerobic exercise. Previous research has demonstrated that both aerobic exercise and WB-EMS have been reported to improve cardiometabolic health indicators in conjunction with favorable body composition changes, and that EMS interventions may influence adipokine profiles and lipid metabolism [43]. The absence of significant changes in HDL-C and TG may reflect differences in the temporal sequence of metabolic adaptations across lipid-related variables.

The decrease in SBP observed in the exercise groups may be attributed to repeated muscle contractions and aerobic stimulation influencing endothelial function and peripheral vascular resistance. Regular exercise has been shown to enhance endothelial nitric oxide synthase activity and nitric oxide production, thereby promoting vasodilation and blood pressure reduction [44,45]. Given that both exercise and WB-EMS have been reported to influence muscle blood flow redistribution, these mechanisms may be associated with the observed reduction in SBP.

Overall, aerobic exercise, with or without WB-EMS, may contribute to improvements in metabolic health through multiple interacting pathways, including enhanced glucose utilization via GLUT4-mediated signaling, improved body composition and fatty acid metabolism, and improved endothelial function. The early improvements observed in LDL-C and FPG, alongside limited changes in HDL-C and TG, suggest that the body’s metabolic adaptation may occur in a staged manner. However, because cellular and molecular markers were not directly measured, future studies should incorporate indices such as HOMA-IR, AS160 (TBC1D4), adiponectin, and nitric oxide levels to further elucidate the metabolic mechanisms underlying WB-EMS-based interventions.

From a practical perspective, the metabolic improvements observed in this study were generally consistent with patterns reported in previous aerobic exercise interventions in obese or sedentary middle-aged populations. Conventional aerobic training has been associated with favorable changes in body composition, glucose regulation, and cardiovascular risk markers. However, because no significant differences were observed between the EXG and WEG, the potential additional contribution of WB-EMS during dynamic movements should be interpreted cautiously, particularly given differences in intervention protocols and participant characteristics across studies. In practical settings, WB-EMS may represent a useful adjunct for individuals who have difficulty performing high-load resistance training because of joint discomfort, obesity-related limitations, or time constraints. However, the implementation of WB-EMS also requires consideration of equipment availability, supervision by trained personnel, and overall accessibility in community or clinical exercise programs.

### 4.4. Integrated Interpretation of Intervention Effects

The concurrent improvements in lower-limb muscle function, FVC-centered pulmonary outcomes, and metabolic health indicators (BMI, WHR, LDL-C, FPG, and SBP) suggest an integrated physiological adaptation rather than isolated changes within a single system. Although improvements were observed across both exercise groups, most muscle function variables did not show significant differences between the EXG and WEG in post hoc comparisons. Therefore, the additional contribution of WB-EMS should be interpreted cautiously and may be limited to specific outcomes such as knee extensor peak torque at 60°/s. Aerobic exercise, with or without WB-EMS, may enhance mitochondrial biogenesis and fatty acid oxidation through Ca^2+^-mediated signaling and the activation of the AMPK–PGC-1α pathway. These adaptations of the body are closely linked to improvements in glucose uptake and insulin sensitivity, consistent with the observed changes in FPG and body composition. Evidence from recent meta-analyses further supports the beneficial effects of WB-EMS on metabolic syndrome risk factors, particularly among physically inactive populations [46].

Alterations in blood flow redistribution and microcirculatory regulation may represent another key mechanism underlying the integrated body adaptation observed. Repeated muscle contractions increase peripheral perfusion and improve oxygen supply–demand matching, thereby stabilizing ATP homeostasis and enhancing contraction efficiency. The progressive increase in exercise intensity and the potential for synchronized motor unit activation induced by WB-EMS may contribute to improvements in muscle perfusion and oxygen utilization, which are consistent with the observed improvements in muscle function and SBP.

In addition, the endocrine function of skeletal muscle may contribute to systemic metabolic regulation. Contraction-induced myokines have been shown to modulate inflammation and enhance insulin sensitivity through activation of AMPK–PGC-1α–GLUT4 signaling pathways, providing a plausible explanation for the observed improvements in LDL-C and FPG relative to HDL-C and TG.

From a respiratory–metabolic coupling perspective, the increase in FVC accompanied by limited changes in FEV_1_ and FEV_1_/FVC suggests that thoracic mobility and auxiliary respiratory muscle coordination may have improved prior to changes in expiratory airflow dynamics. In this context, aerobic exercise, with or without WB-EMS, may represent a practical and efficient intervention that delivers sufficient metabolic stimulus while maintaining low mechanical joint loading, thereby reinforcing positive interactions among the body’s muscular, circulatory, respiratory, and metabolic systems.

Although offering new evidence of the benefits of combining aerobic exercise and EMS, this study has limitations that warrant mention. Primarily, its limitations include its small sample size and its geographic limitation, as it was conducted in a single area in South Korea. In addition, dietary intake was not controlled during the intervention, which may have influenced metabolic outcomes. The use of convenience sampling and the absence of long-term follow-up assessments further limit the generalizability of the findings. As such, caution should be used when generalizing the results to the broader population. However, the findings suggest that aerobic exercise, with or without WB-EMS, can induce integrated physiological adaptations, while the additional benefits of WB-EMS may be limited to specific conditions. These adaptations may involve (1) enhanced muscle metabolism and mitochondrial function, (2) improved microcirculation and oxygen delivery, (3) systemic metabolic regulation mediated by skeletal muscle endocrine function, and (4) strengthened respiratory–metabolic coupling. However, as these mechanisms were not directly assessed, we have interpreted our results as such. Future studies should incorporate electromyography, muscle oxygenation, regional blood flow, myokine and adipokine profiles, nitric oxide signaling, and mitochondrial markers to quantitatively validate these results.

From a practical perspective, WB-EMS may represent a potentially useful adjunct to conventional exercise programs, particularly for individuals who experience difficulty performing high-load resistance training due to joint discomfort, obesity-related limitations, or time constraints. However, the implementation of WB-EMS also involves practical considerations, including equipment costs, the need for trained supervision, and limited availability in many community or clinical settings. No adverse events related to the intervention were reported during the present study, and all sessions were conducted under professional supervision, the safety of WB-EMS should continue to be carefully monitored, particularly in populations with underlying cardiovascular or metabolic conditions. Future research should further evaluate the long-term safety, cost-effectiveness, and accessibility of WB-EMS-based exercise interventions in real-world settings. However, because most outcomes did not demonstrate significant between-group differences, the present findings do not provide strong evidence for additional benefits of WB-EMS beyond aerobic exercise alone.

## 5. Conclusions

This study examined the effects of aerobic exercise combined with WB-EMS on muscle function, pulmonary function, and metabolic health indicators in obese middle-aged women. Compared with aerobic exercise alone, the WB-EMS combined exercise program produced greater improvements in lower-limb muscle strength, particularly in knee extensor function, while changes in high-velocity muscle performance were less distinct. Pulmonary outcomes showed a significant increase in FVC, whereas expiratory flow indices remained unchanged, suggesting partial and indirect respiratory adaptations. However, there were favorable changes in several metabolic health indicators, including body composition-related indices, FPG, and SBP. Improvements in glucose regulation tended to be greater in the WB-EMS group, although no significant additional metabolic benefits of WB-EMS compared with aerobic exercise alone were observed. Observed associations between muscle function and metabolic or pulmonary variables may indicate that skeletal muscle adaptations could be related to broader systemic responses. Overall, aerobic exercise, with or without WB-EMS, appears to support improvements in musculoskeletal and metabolic health in obese middle-aged women. WB-EMS may serve as a potential adjunct to conventional aerobic training; however, its additional benefits beyond aerobic exercise alone appear to be limited based on the present findings. However, because this study involved a modest sample size and a relatively short intervention period, larger randomized trials with longer follow-up periods are needed to confirm these findings and further clarify the underlying physiological mechanisms.

## Figures and Tables

**Table 1:** Overview of the 12-week aerobic exercise program with and without WB-EMS.

Phase	Period	Exercises		Execution	Intensity	WB-EMS
Warm-up (10 min)	Entire period	Joint mobilization and dynamic stretching		Whole-body joint movements with low-intensity rhythmic actions	Low	Not applied
Main exercise (20 min)	Weeks 1–4 (level 1)	Basic aerobic movements (march, knee lift, lunge, jumping jack, skip, running, kick)	Continuous aerobic movements synchronized with music Each movement: 30–60 s Rest between movements: 15–30 s Repetition of 7 movement patterns	HRR 35–45%	EXG: not applied WEG: 30–40 Hz	
	Weeks 5–8 (level 2)			HRR 45–60%	EXG: not applied WEG: 50–60 Hz	
	Weeks 9–12 (level 3)			HRR 60–70%	EXG: not applied WEG: 65–80 Hz	
Cool-down (10 min)	Entire period	Muscle relaxation and static stretching		Static stretching of major muscle groups	Low	Not applied

WB-EMS, whole-body electromyostimulation; HRR: heart rate reserve; EXG: aerobic exercise group without WB-EMS; WEG: aerobic exercise group combined with WB-EMS.

**Table 2:** Baseline characteristics of the study participants.

Variables			CON (*n* = 12)	EXG (*n* = 12)	WEG (*n* = 12)	F	*p*
Age (years)			43.25 ± 3.05	45.17 ± 3.51	45.17 ± 3.33	1.349	0.274
Anthropometric characteristics	Height (cm)		157.00 ± 4.86	158.08 ± 3.60	156.83 ± 4.11	0.310	0.736
	Weight (kg)		62.68 ± 3.68	65.72 ± 3.64	64.48 ± 2.92	2.385	0.108
Muscle function	60°/s peak torque (Nm)	Right knee extension	130.88 ± 1.42	129.77 ± 2.80	130.68 ± 1.91	0.932	0.404
		Right knee flexor	72.28 ± 1.49	71.30 ± 1.14	71.26 ± 1.35	2.279	0.118
		Left knee extension	128.16 ± 2.41	127.91 ± 2.39	128.46 ± 3.06	0.131	0.878
		Left knee flexor	72.04 ± 1.24	71.56 ± 1.36	71.73 ± 1.14	0.459	0.636
	180°/s average power (W)	Right knee extension	117.83 ± 2.48	118.67 ± 1.72	117.83 ± 2.04	0.628	0.540
		Right knee flexor	52.08 ± 3.03	51.67 ± 2.71	51.17 ± 3.07	0.292	0.748
		Left knee extension	117.00 ± 2.00	117.00 ± 2.26	117.00 ± 2.26	0.000	1.000
		Left knee flexor	48.00 ± 2.09	48.75 ± 3.05	46.83 ± 1.80	1.987	0.153
	180°/s total work (J)	Right knee extension	514.25 ± 30.85	517.42 ± 29.98	492.33 ± 21.19	2.921	0.068
		Right knee flexor	298.75 ± 22.17	282.33 ± 20.99	288.33 ± 15.73	2.106	0.138
		Left knee extension	446.08 ± 41.71	468.92 ± 25.90	453.83 ± 30.36	1.456	0.248
		Left knee flexor	276.58 ± 12.85	288.17 ± 17.24	273.33 ± 23.27	2.181	0.129
Pulmonary function	Forced vital capacity (L)		2.89 ± 0.10	2.85 ± 0.12	2.90 ± 0.07	1.060	0.358
	Forced expiratory volume in one second (L)		2.32 ± 0.09	2.29 ± 0.15	2.22 ± 0.11	1.977	0.155
	Forced expiratory volume in 1 s to forced vital capacity ratio (%)		73.84 ± 3.21	72.87 ± 5.50	73.19 ± 2.48	0.190	0.828
Metabolic health indicators	Body mass index (kg/m^2^)		26.80 ± 1.18	27.94 ± 1.56	27.27 ± 1.61	1.849	0.173
	Percent body fat (%)		29.20 ± 2.06	30.49 ± 2.48	31.84 ± 2.65	3.613	0.038
	Waist-to-hip ratio		0.87 ± 0.02	0.87 ± 0.02	0.87 ± 0.02	0.183	0.834
	Triglycerides (mg/dL)		172.25 ± 83.00	187.67 ± 113.08	146.00 ± 79.35	0.615	0.547
	High-density lipoprotein cholesterol (mg/dL)		49.50 ± 11.38	52.17 ± 13.54	42.33 ± 10.71	2.177	0.129
	Low-density lipoprotein cholesterol (mg/dL)		138.42 ± 31.20	135.92 ± 22.12	146.75 ± 27.90	0.517	0.601
	Fasting plasma glucose (mg/dL)		116.67 ± 5.77	115.25 ± 5.28	116.17 ± 5.11	0.213	0.809
	Systolic blood pressure (mmHg)		147.17 ± 8.53	135.25 ± 11.37	138.08 ± 13.18	3.714	0.035
	Diastolic blood pressure (mmHg)		84.50 ± 13.51	81.42 ± 9.62	79.42 ± 6.14	0.754	0.478

Values are presented as mean ± standard deviation. CON: control group; EXG: aerobic exercise group; WEG: aerobic exercise group combined with whole-body electromyostimulation.

**Table 3:** Comparisons of lower-limb isokinetic muscle strength, power, and endurance before and after the 12-week intervention in the three groups.

Variables		Group	Pre	Post	Effect	F	*p*	Post Hoc
60°/s peak torque (Nm, %BW)	Right knee extensor	CON (*n* = 12)	130.88 ± 1.42	131.02 ± 1.07	Group (A)	4.498	0.019 *	a = b < c
		EXG (*n* = 12)	129.77 ± 2.80	134.16 ± 3.64	Time (B)	46.570	<0.001 ***	
		WEG (*n* = 12)	130.68 ± 1.91	136.20 ± 3.21	A × B	11.122	<0.001 ***	
	Left knee extension	CON (*n* = 12)	128.16 ± 2.41	128.08 ± 3.69	Group (A)	10.319	<0.001 ***	a < b < c
		EXG (*n* = 12)	127.91 ± 2.39	134.41 ± 2.51	Time (B)	85.737	<0.001 ***	
		WEG (*n* = 12)	128.46 ± 3.06	136.93 ± 3.55	A × B	23.216	<0.001 ***	
	Right knee flexor	CON (*n* = 12)	72.28 ± 1.49	72.01 ± 1.59	Group (A)	4.005	0.028 *	a < b = c
		EXG (*n* = 12)	71.30 ± 1.14	76.07 ± 4.15	Time (B)	31.685	<0.001 ***	
		WEG (*n* = 12)	71.26 ± 1.35	77.04 ± 3.83	A × B	9.477	0.001 **	
	Left knee flexor	CON (*n* = 12)	72.04 ± 1.24	72.08 ± 1.31	Group (A)	5.741	0.007 **	a < b = c
		EXG (*n* = 12)	71.56 ± 1.36	75.83 ± 3.37	Time (B)	44.363	<0.001 ***	
		WEG (*n* = 12)	71.73 ± 1.14	76.72 ± 3.14	A × B	11.047	<0.001 ***	
180°/s average power (W, %BW)	Right knee extensor	CON (*n* = 12)	117.83 ± 2.48	117.50 ± 2.11	Group (A)	3.539	0.040 *	a = b = c
		EXG (*n* = 12)	118.67 ± 1.72	120.58 ± 1.78	Time (B)	38.737	<0.001 ***	
		WEG (*n* = 12)	117.83 ± 2.04	120.83 ± 2.25	A × B	15.994	<0.001 ***	
	Left knee extension	CON (*n* = 12)	117.00 ± 2.00	116.33 ± 2.47	Group (A)	0.907	0.414	a = b = c
		EXG (*n* = 12)	117.00 ± 2.26	118.17 ± 2.04	Time (B)	4.439	0.043 *	
		WEG (*n* = 12)	117.00 ± 2.26	118.42 ± 2.50	A × B	4.690	0.016 **	
	Right knee flexor	CON (*n* = 12)	61.25 ± 3.36	61.17 ± 4.22	Group (A)	0.616	0.546	-
		EXG (*n* = 12)	60.33 ± 2.46	60.83 ± 4.02	Time (B)	1.776	0.192	
		WEG (*n* = 12)	59.08 ± 1.73	60.75 ± 3.02	A × B	0.975	0.388	
	Left knee flexor	CON (*n* = 12)	57.67 ± 2.77	57.83 ± 2.44	Group (A)	0.356	0.703	-
		EXG (*n* = 12)	56.58 ± 2.35	57.67 ± 2.39	Time (B)	3.651	0.065	
		WEG (*n* = 12)	56.50 ± 2.24	57.83 ± 2.41	A × B	0.619	0.544	
180°/s total work (J)	Right knee extensor	CON (*n* = 12)	514.25 ± 30.85	516.67 ± 32.29	Group (A)	0.585	0.563	-
		EXG (*n* = 12)	517.42 ± 29.98	530.50 ± 35.74	Time (B)	9.869	0.004 **	
		WEG (*n* = 12)	492.33 ± 21.19	527.58 ± 55.53	A × B	2.945	0.067	
	Left knee extension	CON (*n* = 12)	446.08 ± 41.71	472.00 ± 25.87	Group (A)	0.721	0.494	-
		EXG (*n* = 12)	468.92 ± 25.90	468.42 ± 37.18	Time (B)	2.556	0.119	
		WEG (*n* = 12)	453.83 ± 30.36	463.50 ± 26.71	A × B	1.106	0.343	
	Right knee flexor	CON (*n* = 12)	298.75 ± 22.17	297.00 ± 19.41	Group (A)	1.045	0.363	-
		EXG (*n* = 12)	282.33 ± 20.99	292.67 ± 18.97	Time (B)	6.557	0.015 *	
		WEG (*n* = 12)	288.33 ± 15.73	296.42 ± 13.93	A × B	2.924	0.068	
	Left knee flexor	CON (*n* = 12)	276.58 ± 12.85	272.00 ± 18.31	Group (A)	2.480	0.099	a = b = c
		EXG (*n* = 12)	288.17 ± 17.24	290.50 ± 18.74	Time (B)	1.352	0.253	
		WEG (*n* = 12)	273.33 ± 23.27	283.92 ± 18.94	A × B	3.367	0.047 *	

CON: control group; EXG: aerobic exercise group without whole-body electromyostimulation; WEG: aerobic exercise group combined with whole-body electromyostimulation; %BW: percent body weight. a = CON, b = EXG, c = WEG. * *p* < 0.05, ** *p* < 0.01, *** *p* < 0.001; tested using a two-way repeated-measures analysis of variance (group × time), followed by Bonferroni post hoc tests where appropriate.

**Table 4:** Comparisons in pulmonary function before and after the 12-week intervention in obese middle-aged women.

Variables	Group	Pre	Post	Effect	F	*p*	Post-Hoc
Forced vital capacity (L)	CON (*n* = 12)	2.89 ± 0.10	2.86 ± 0.07	Group (A)	3.054	0.061	a = b = c
	EXG (*n* = 12)	2.85 ± 0.12	3.02 ± 0.08	Time (B)	13.807	<0.001 ***	
	WEG (*n* = 12)	2.90 ± 0.07	2.97 ± 0.09	A × B	9.393	<0.001 ***	
Forced expiratory volume in one second (L)	CON (*n* = 12)	2.32 ± 0.09	2.32 ± 0.09	Group (A)	2.951	0.066	-
	EXG (*n* = 12)	2.29 ± 0.15	2.27 ± 0.09	Time (B)	0.025	0.876	
	WEG (*n* = 12)	2.22 ± 0.11	2.24 ± 0.07	A × B	0.347	0.709	
Forced expiratory volume in one second to forced vital capacity ratio (%)	CON (*n* = 12)	73.84 ± 3.21	74.15 ± 2.03	Group (A)	0.297	0.745	-
	EXG (*n* = 12)	72.87 ± 5.50	73.08 ± 3.84	Time (B)	0.618	0.438	
	WEG (*n* = 12)	73.19 ± 2.48	73.46 ± 2.47	A × B	0.008	0.992	

CON: control group; EXG: aerobic exercise group without whole-body electromyostimulation; WEG: aerobic exercise group combined with whole-body electromyostimulation. a = CON, b = EXG, c = WEG. *** *p* < 0.001; tested using a two-way repeated-measures analysis of variance (group × time), followed by Bonferroni post hoc tests where appropriate.

**Table 5:** Comparisons in body composition, blood lipid profile, glycemic control, and blood pressure before and after the 12-week intervention.

Variables	Group	Pre	Post	Effect	F	*p*	Post-Hoc
Body mass index (kg/m^2^)	CON (*n* = 12)	26.80 ± 1.18	26.82 ± 1.17	Group (A)	1.676	0.203	-
	EXG (*n* = 12)	27.94 ± 1.56	27.69 ± 1.33	Time (B)	6.677	0.014 *	
	WEG (*n* = 12)	27.27 ± 1.61	27.05 ± 1.38	A × B	2.088	0.140	
Body fat (%)	CON (*n* = 12)	29.20 ± 2.06	29.68 ± 2.56	Group (A)	2.691	0.083	-
	EXG (*n* = 12)	30.49 ± 2.48	30.21 ± 2.22	Time (B)	0.133	0.718	
	WEG (*n* = 12)	31.84 ± 2.65	31.46 ± 2.42	A × B	2.392	0.107	
Waist-to hip ratio	CON (*n* = 12)	0.87 ± 0.02	0.87 ± 0.02	Group (A)	0.869	0.429	-
	EXG (*n* = 12)	0.87 ± 0.02	0.86 ± 0.02	Time (B)	8.001	0.008 **	
	WEG (*n* = 12)	0.87 ± 0.02	0.86 ± 0.02	A × B	0.615	0.546	
Triglycerides (mg/dL)	CON (*n* = 12)	172.25 ± 82.99	151.17 ± 40.63	Group (A)	1.780	0.184	-
	EXG (*n* = 12)	187.67 ± 113.08	145.75 ± 50.36	Time (B)	4.129	0.050	
	WEG (*n* = 12)	146.00 ± 79.35	111.75 ± 25.92	A × B	0.145	0.865	
High-density lipoprotein cholesterol (mg/dL)	CON (*n* = 12)	49.50 ± 11.38	52.42 ± 7.82	Group (A)	1.738	0.192	-
	EXG (*n* = 12)	52.17 ± 13.54	50.92 ± 9.78	Time (B)	1.409	0.244	
	WEG (*n* = 12)	42.33 ± 10.71	49.92 ± 12.09	A × B	0.964	0.392	
Low-density lipoprotein cholesterol (mg/dL)	CON (*n* = 12)	138.42 ± 31.20	146.83 ± 24.21	Group (A)	0.184	0.833	a = b = c
	EXG (*n* = 12)	135.92 ± 22.12	139.00 ± 11.63	Time (B)	0.292	0.593	
	WEG (*n* = 12)	146.75 ± 27.90	130.50 ± 19.36	A × B	6.537	0.004 **	
Fasting plasma glucose (mg/dL)	CON (*n* = 12)	116.67 ± 5.77	118.08 ± 4.17	Group (A)	1.366	0.269	a = b = c
	EXG (*n* = 12)	115.25 ± 5.28	116.92 ± 3.20	Time (B)	0.082	0.776	
	WEG (*n* = 12)	116.17 ± 5.11	112.50 ± 5.73	A × B	6.542	0.004 **	
Systolic blood pressure (mmHg)	CON (*n* = 12)	147.17 ± 8.53	146.75 ± 7.25	Group (A)	5.169	0.011 *	-
	EXG (*n* = 12)	135.25 ± 11.37	134.42 ± 9.00	Time (B)	6.020	0.020 *	
	WEG (*n* = 12)	138.08 ± 13.18	134.67 ± 10.92	A × B	2.190	0.128	
Diastolic blood pressure (mmHg)	CON (*n* = 12)	84.50 ± 13.51	85.58 ± 8.99	Group (A)	1.170	0.323	-
	EXG (*n* = 12)	81.42 ± 9.62	81.67 ± 9.61	Time (B)	0.395	0.534	
	WEG (*n* = 12)	79.42 ± 6.14	81.00 ± 5.13	A × B	0.063	0.939	

CON: control group; EXG: aerobic exercise group without whole-body electromyostimulation; WEG: aerobic exercise group combined with whole-body electromyostimulation. a = CON, b = EXG, c = WEG. * *p* < 0.05, ** *p* < 0.01; tested using a two-way repeated-measures analysis of variance (group × time), followed by Bonferroni post hoc tests where appropriate.

## Data Availability

The data presented in this study are available on request from the corresponding author due to ethical restrictions and the inclusion of sensitive participant information.
